# Refining discordant gene trees

**DOI:** 10.1186/1471-2105-15-S13-S3

**Published:** 2014-11-13

**Authors:** Pawel Górecki, Oliver Eulenstein

**Affiliations:** 1 Institute of Informatics, University of Warsaw, Banacha 2, 02-097 Warsaw, Poland; 2 Department of Computer Science, Iowa State University, Atanasoff Hall 212, 50011 Ames, USA

**Keywords:** Gene trees, Discordance, Tree comparison cost, Robinson-Foulds cost, gene duplication cost, deep coalescence cost

## Abstract

**Background:**

Evolutionary studies are complicated by discordance between gene trees and the species tree in which they evolved. Dealing with discordant trees often relies on comparison costs between gene and species trees, including the well-established Robinson-Foulds, gene duplication, and deep coalescence costs. While these costs have provided credible results for binary rooted gene trees, corresponding cost definitions for non-binary unrooted gene trees, which are frequently occurring in practice, are challenged by biological realism.

**Result:**

We propose a natural extension of the well-established costs for comparing unrooted and non-binary gene trees with rooted binary species trees using a binary refinement model. For the duplication cost we describe an efficient algorithm that is based on a linear time reduction and also computes an optimal rooted binary refinement of the given gene tree. Finally, we show that similar reductions lead to solutions for computing the deep coalescence and the Robinson-Foulds costs.

**Conclusion:**

Our binary refinement of Robinson-Foulds, gene duplication, and deep coalescence costs for unrooted and non-binary gene trees together with the linear time reductions provided here for computing these costs significantly extends the range of trees that can be incorporated into approaches dealing with discordance.

## Introduction

*Gene trees *represent estimates of evolutionary histories of gene families, and are fundamental for evolutionary biological research [1,2]. Often gene trees are assumed to reflect the evolutionary history of species, or *species tree*, from which their sequences were sampled, presenting a common approach of species tree inference [3-7]. Gene trees can also provide fundamental information to study the evolution of biochemical function in gene families [8].

Gene trees can be inferred from multiple sequence alignments of sequences culled from a gene family. The number of these sequences as well as their evolutionary complexity has expanded on an unprecedented scale in recent years [9], prompting the estimation of ever larger and more credible gene trees. Despite these potentials, evolutionary biologists have long recognized the potential for substantial discordance among the gene trees as well as among the gene trees and the species tree in which they evolve [10-14], challenging traditional phylogenetic gene tree and species tree estimation. Discordance can be caused by error as well as major evolutionary processes, such as the duplication of genes or deep coalescence. Complicating matters further such error and evolutionary processes can occur on a staggering scale [15,16]. For example simulations with realistic parameters suggested that analyzes individual avian genes frequently resulted in trees with substantial error [17], and evolutionary processes cause discordance among evolutionary relationships of major avian groups [18]. Consequently, phylogenetic approaches are challenged to deal with error as well as complex histories of evolutionary processes in order to explain discordance in gene trees [19-21].

A common approach to deal with discordance in gene trees is by representing them with an estimate of the species tree that is thought to be the median tree of the gene trees under a particular *(topological comparison) cost *from a gene tree to a species tree, which is often referred to as a *supertree *[22]. A *median tree S *for a given cost and a collection of trees minimizes the sum of the pairwise costs from every gene tree to *S*. While varies costs have been proposed [23-26], here we are concerned with the well-researched Robinson-Foulds, duplication, and deep coalescence costs. The Robinson-Foulds cost is measuring quantitative dissimilarities between two trees without relying on an evolutionary model, and is therefore well suited to address discordance caused by error [27,28]. In difference, the costs for the evolutionary events gene duplication and deep coalescence are both based on an evolutionary parsimony model allowing to resolve discord based on such events [29,30].

However, the presented costs are not well adapted to biological realism [31,32]. In practice gene trees are frequently inferred from sequences that do not permit reliable estimations of rootings or bifurcations [33], and therefore are unrooted and non-binary. The original evolutionary costs for gene duplication and deep coalescence can not be applied to such trees, since they are only defined for rooted and binary gene trees. In contrast the Robinson-Foulds distance is formally defined for unrooted and non-binary trees, but multifurcations in phylogenetic trees are interpreted as true evolutionary multifurcations (*hard multifurcations*). However, non-binary relationships in gene trees represent uncertainties about the correct binary relationships (*soft multifurcations*), rather than hard multifurcations which are rare [34]. Consequently, all of the the presented costs are not applicable to a large number of gene trees in practice.

More recently, a binary refinement model for the duplication cost [35] and the deep coalescence cost [36,37] for rooted gene trees that are non-binary were introduced. Here we propose a natural extension of this model for our costs to compare unrooted and non-binary gene trees with rooted binary species trees, and describe linear time reductions to compute these costs.

## Related work

Here we provide definitions as well as computational and applicability results, first for the Robinson-Foulds cost, and then for the duplication and deep coalescence costs.

The Robinson-Foulds cost is an elementary tool for estimating quantitative dissimilarities between phylogenetic trees [38-40]. This cost is defined for two trees to be the cardinality of the symmetric difference of their split presentations for unrooted trees, and of their cluster presentations for rooted trees. The *split-presentation *of an unrooted tree is the set of all bipartitions, called *splits*, of the trees' taxon set induced by the removal of an edge [39,41]. Analogously, the *cluster presentation *of a rooted tree is the set of all taxon sets of its full subtrees [39]. The Robinson-Foulds cost for two trees, both either unrooted or rooted, satisfies the metric properties [38], and can be computed in linear time [42]. A randomized approximation scheme computes, in sublinear time and with high probability, a (1 + ∈) approximation of the Robinson-Foulds cost [43]. More recently, the Robinson-Foulds cost between an unrooted tree and a rooted tree was introduced in [44] to be the minimum cost under all pairs consisting of a rooting of the unrooted tree and the rooted tree. In fact, this cost is still computable in linear time [44]. Moreover, the distribution of the Robinson-Foulds distance relative to a fixed tree can be computed in linear time [45]. Note, the skewed distribution of the Robinson-Foulds metric suggests that it is only of use when the trees to be compared are quite similar [46]. While the Robinson-Foulds cost is wide-spread for the comparative analysis of phylogenetic trees, it does not rely on a biological model explaining the difference between trees. Therefore, the Robinson-Foulds cost is generally applicable to any type of trees, e.g. linguistic trees [47] and trees representing dominance hierarchies [48].

In contrast, the duplication and the deep coalescence costs rely on a biological model explaining the discordance between a gene tree and a species tree based on evolutionary events. For a gene and a species tree, both rooted and binary, the *duplication cost *and the *deep coalescence cost *are defined to be the minimum number of gene duplications and coalescences, respectively, required to reconcile the gene tree with the species tree [49,50]. While theses costs are not symmetric, they are computable in linear time [51,52], and allow to infer credible species trees [53-57]. Furthermore, gene trees that are reconciled by the minimum number of evolutionary events allow studying complex histories of evolutionary events [54,58]. The gene duplication and deep coalescence costs can also be defined for binary unrooted gene trees and binary rooted species trees as the minimum cost under all rootings of the gene tree and computed in linear time [32,59,60]. However, often gene trees are unrooted and non-binary in practice. While existing definitions for such gene trees and rooted binary species trees are linear time computable [31,32], they are not well adapted to biological realism. More recently, cost definitions for such trees were introduced that are based on a binary refinement model, by choosing the minimum cost between every binary refinement of a rooted gene tree and a rooted binary species tree, which are polynomial time computable [35,61]. In contrast, finding the minimum cost between a rooted binary gene tree and all binary refinements of a rooted non-binary species tree is NP hard [37]. However, costs under a binary refinement model for unrooted and non-binary gene trees have not been addressed in the literature. For a detailed overview about gene tree reconciliation the interested reader is referred to [62].

### Contributions

Here, we define the Robinson-Foulds, duplication, and deep coalescence costs for unrooted and non-binary gene trees and a rooted binary species tree under the binary refinement model. To compute the duplication cost we describe a linear time reduction from the problem of computing optimal binary refinements of unrooted gene trees to the problem of computing such refinements for rooted gene trees. The latter problem can be solved in linear time [37]. Then, based on the theory of unrooted tree reconciliation [32,44,63,59], we prove that the duplication cost has similar properties to the deep coalescence and Robinson-Foulds costs when comparing unrooted and non-binary gene trees with rooted species trees. From this follows that we can prove linear time reductions for the deep coalescence and the Robinson-Foulds costs that are similar to our reduction for the duplication cost. Since our reductions require only linear time, the runtime to compute the optimal binary refinements of unrooted gene trees is bound by the time complexity of computing optimal binary refinement for rooted binary gene trees.

## Basic definitions and preliminaries

An *unrooted tree T *is an acyclic, connected, and undirected graph that has no degree-two nodes, and every degree-one node is labeled with a species name. The degree-one nodes are called *leaves*; and the remaining nodes are called *internal *nodes. A tree is binary if every internal node has degree three. A *rooted *tree is defined similar to an unrooted tree, with the difference that it has a distinguished node, called *root. A contraction *of an edge *e *of an (un)rooted tree *T *removes *e *from *T *and merges both ends of *e *into a single node. *A binary refinement *of an unrooted or rooted tree *T *is a binary tree that can be transformed into *T *by contractions. By *L*(*T *) we denote the set of all leaf labels in *T *.

A rooted tree *S *with a unique leaf labeling is called *a species tree*. For two nodes *a, b *of *S, a *⊕ *b *is the least common ancestor of *a *and *b *in *S*. Let *T *and be a rooted tree (called rooted gene tree) such that *L*(*T *) ⊆ *L*(*S*). By *M *: *T → S *we denote the *least common ancestor (lca) mapping *between the nodes of *T *and *S *that preserves the labeling of the leaves. The *duplication cost *between *T *and *S*, is defined by: *D*(*T, S*) := |{*M *(*g*) = *M *(*c*) : *c *is a child of an internal node *g *∈ *T *}|.

Let *G *= 〈*V_G_, E_G_*〉 be an unrooted tree (called unrooted gene tree). A *rooting *of *G *is defined by choosing an edge *e *from *G *on which the root is to placed. Such a rooted tree will be denoted by *G_e_*. Note that *G_e _*has one more node (the root) that *G*. A *rooted binary refinement *of an unrooted gene tree *G*, is a binary refinement of a rooting of *G*.

The unrooted *duplication (urD) *cost between an unrooted gene tree *G *and a species tree *S *is defined as

urD(G, S) := min{D(G!, S) :G! is a rooting of G}.

The edges with minimal cost will be called optimal. In the remainder of this work we show first how to compute *urD *in linear time and space, and then solve the following problem. Observe, that in contrast to our previous study [44,32,64], here, for the first time, we extend the notion of rooting by incorporating rooting at nodes.

**Problem 1 ***For a given unrooted gene tree G and a binary species tree S, find the binary refinement of under all rootings of G that minimizes the duplication cost*.

A similar problem for rooted gene trees was solved in [35]. In the remaining section we show how to reduce Problem 1 to the rooted problem in linear time.

### Unrooted reconciliation

**Figure 1:**
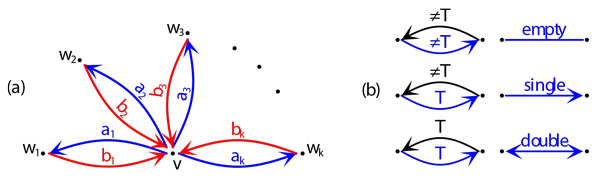
**Star transformation**. (a) A star with the center *v *in $\hat{G}$ and *k ≥ *3 edges. Here *ei *= {*v, w_i_*} for *i *= 1, 2*, . . . , k*. (b) A simplified representation of edges (empty, single and double) that will be used through the rest of this work. The notation ≠ ⊤ denotes that the label is a non-root node from *S*.

First we provide definitions introducing the basics of unrooted reconciliation. This approach is partially based on our previous papers [32,44,63,59]. However, for the first time, we prove properties of *urD *for trees with multifurcations. We assume that *G *is an unrooted gene tree and *S *is a species tree. We transform *G *into a directed graph $\hat{G}$, by replacing each edge {*v, w*} by a pair of directed edges 〈*v, w*〉 and 〈*w, v*〉. We label the edges of $\hat{G}$ by the nodes of *S *as follows. If *v *∈ *G *is a leaf labeled by *a*, then the edge {*v, w*} in $\hat{G}$ is labeled by the node in *S *whose label is *a*. Let *v *∈ *G *have exactly *k *siblings *w*_1_*, w*_2_*, . . . , w_k_*. If *a_i _*and *b_i _*are the labels of 〈*v, w_i_*〉 and 〈*w_i_, v*〉, respectively, then $ai={⊕}_{j=1,\;j\neq i}^{j=k}\;b_{j}$. Let ⊤ be the root of *S*. Each internal node *v *∈ *G *defines a *star *with the center *v *as indicated in Figure 1a. We refer to the undirected edge {*v, w_i_*} as *e_i_*, for all *i *= 1, 2*, . . . k*.

There is a limited number of star types in gene trees [44]. Let *K *be a star with center *v *and *k *siblings as indicated in Figure 1a. Let *α *denote the number of edges satisfying *a_i _*= ⊤. Similarly, we define *β *for *bi*'s. Then, *K *has type: **M1 **if *α *= 1 and *β *= *k − *1 and all edges labeled by ⊤ are connected to the *k *siblings of *v*, **M2 **if *α *= 0 and *β *= *k − *1, **M3 **if *α *= 1 and *β *= *k*, **M4 **if *α *= *β *= *k*, **M5 **if 1 *< α < β *= *k *and **M6 **if *α *= 0 and *β *= *k*.

**Proposition 1 ***For a given unrooted gene tree G and a species tree S a gene tree G can have any number of stars M1. For the remaining stars we have three mutually exclusive cases: (i) G has an empty edge, (ii) G has a double edge or (iii) G has only single edges*.

*Proof *The proof follows easily from the properties of stars. See also Lemma 2 from [44].   □

Observe that in case (i) *G *has one or two stars M2, in case (ii) *G *has a star of type M3-M5 and in (iii) *G *has exactly one star of type M6.

The next propositions states a crucial difference between binary and general trees. For the proof please refer to [44].

**Proposition 2 ***If both an unrooted gene tree G and a species S are binary then G has at least one empty or double edge*.

## Results

### Polytomies and the duplication cost

The next two proposition shows how the cost changes when we move a position of the root in *G*.

**Proposition 3 ***Under the notation from *Figure 1. *If for some i *∈ {1, 2*, . . . , k*} *one of the following conditions are true:*

• *If the star type is M1 or M3 and b_i _*= ⊤.

• *If the star type is M2 and a_i _≠ *⊤ *≠ b_i_*.

*then $D\left(G_{e_{i}},S\right)\leq D\left(G_{e_{j}},S\right)$for every j *= 1, 2*, . . . , k*.

**Figure 2:**
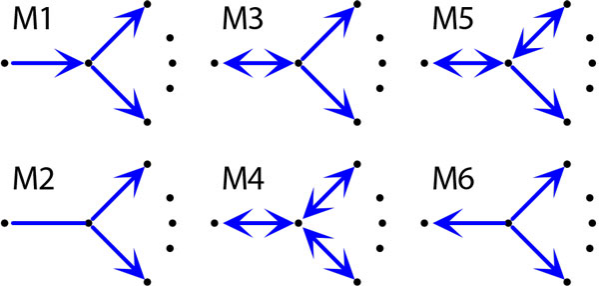
**Stars**. Star topologies that can be present in gene trees. On the right side of stars there are at least 2 edges. M5 has at least two double edges and at least one single edge.

*Proof *All rootings of *G *share the same subtrees attached to *w*_1_*, w*_2_*, . . . w_k_*. Therefore, all costs share the same component *c *coming from the partial duplication cost for these subtrees. The remainder follows in from the definition of the duplication cost and Figure 1 and Figure 2. For *l *∈ {1, 2*, . . . k*} let *M_l _*be the lca-mapping from $G_{e_{i}}$ to *S*. In the case of stars M1 or M3 we have *M_j _*(*v*) = *M_j _*(*w_i_*) = ⊤. Therefore, both nodes, *w *and the root of $G_{e_{j}}$, are duplication nodes; that is, $D\left(G_{e_{j}},S\right)=c+2$. However, in $G_{e_{i}}$, *v *can be a non-duplication node, thus $c+1\leq D\left(G_{e_{i}},S\right)\leq D\left(G_{e_{j}},S\right)=c+2$.

In the case of M2, we have *M_i_*(*w_i_*) ≠ ⊤ ≠ *M_i_*(*v*) and *M_i_*(*w_i_*) ⊕ *M_i_*(*v*) = ⊤, thus the root of *G_i _*is a non-duplication node. On the other hand, *M_j_*(*v*) = ⊤ and the root of *G_j _*is a duplication node. We conclude $c\leq D\left(G_{e_{i}},S\right)\leq c+1\leq D\left(G_{e_{j}},S\right)$.    □

**Proposition 4 ***Using the notation from Proposition 3. If the star type is M*4 *− M*6 *then $D\left(G_{e_{i}},S\right)=D\left(G_{e_{j}},S\right)$ for all i and j*.

*Proof *Similarly to the proof of the previous proposition, it is easy to show that the root of *G_ei _*is is a duplication node while *v *is a duplication node, if and only if, the star is of type M4 or M5. Therefore, for every *i*, $D\left(G_{e_{i}},S\right)=c+1$ if the star type is M6 and $D\left(G_{e_{i}},S\right)=c+2$. Otherwise, where *c *is defined in the proof of Proposition 3.    □

We conclude from Propositions 1-4:

**Theorem 1 ***For an unrooted gene tree G and a species tree S. If e is an edge of G that is either empty, double or an element of a star M6, then e is optimal*.

This observation leads to a linear time and space reduction for *urD *computation similar to algorithms from [32,44]. Now we reduce Problem 1, to the problem where gene trees are rooted. In the special case of star M6, we need to root a tree at a node instead of edge. For a non-leaf node *v *∈ *VG *by *Gv *we denote the tree rooted at *v*. We refer to the algorithm for refining rooted gene trees from [65] by *Bin*(*T, S*), where *T *is a rooted tree and *S *is a binary species tree. It is known that *Bin*(*T, S*) runs in *O*(*|T ||S|*) time [65].

**Theorem 2 ***Algorithm 1 infers a rooted binary refinement G∗ of an unrooted gene tree G such that D*(*G∗, S*) = min *{urD*(*G', S*) : *G' is a binary refinement of G*}.

**Figure 3:**
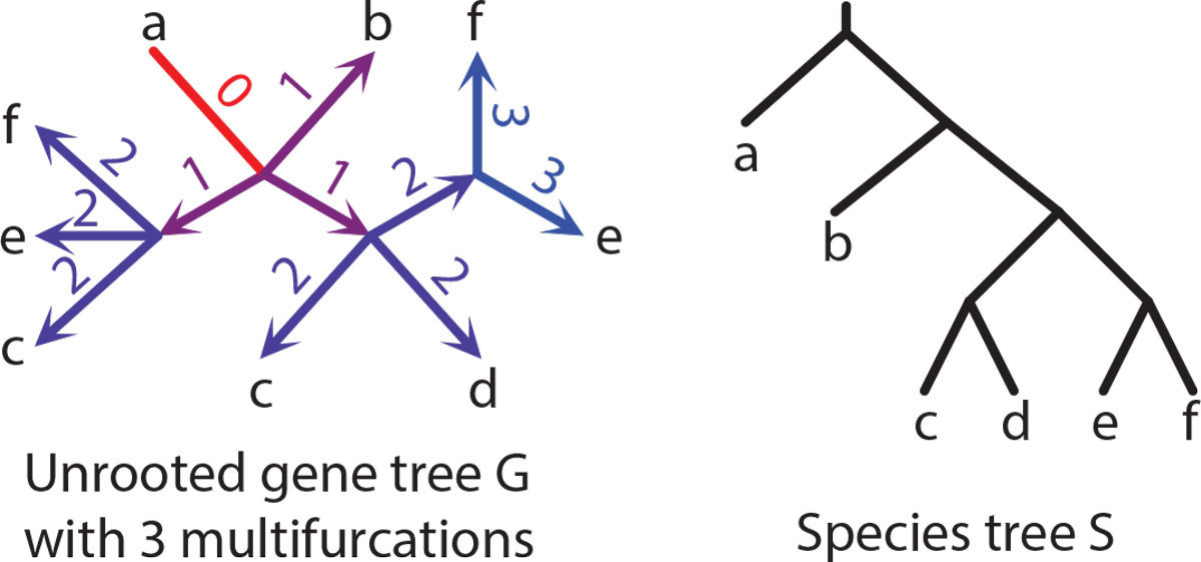
**Gene tree and species trees**. An example of an unrooted gene tree *G *with three multifurcations and a species tree *S*. The gene tree *G *is depicted with a star topology, and it has one star of type M2 and three stars of type M1. Every edge *e *of *G *is decorated with the duplication cost *D*(*G_e_, S*) (note that the rooting *G_e _*is not refined). Observe, that the optimal edge (empty edge) is adjacent to a leaf labelled by *a*. Rooting at this edge yields the duplication cost 0.

**Figure 4:**
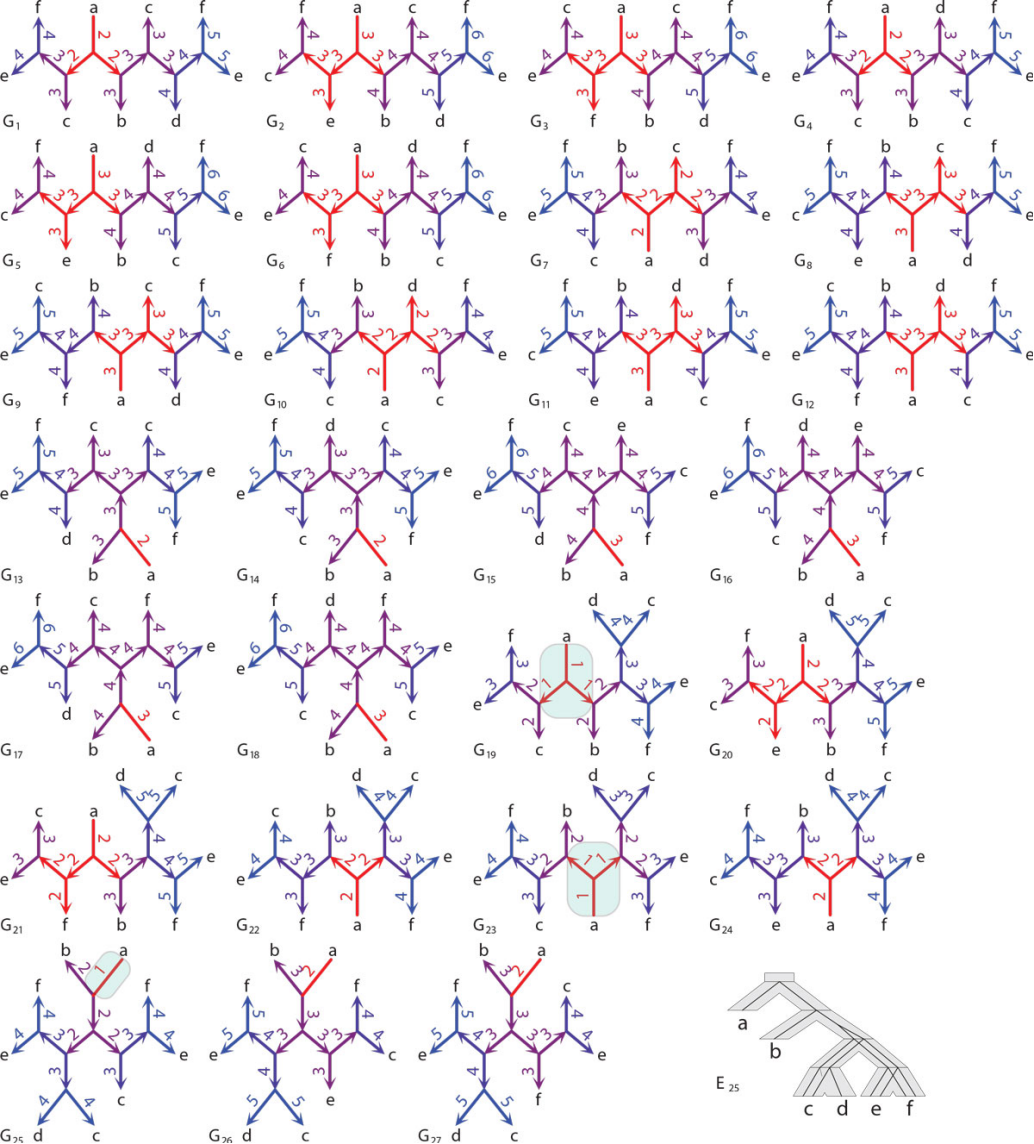
**Binary refinements and unrooted reconciliation**. All 27 unrooted binary refinements of the gene tree *G *from Figure 3 shown in star-like topology. Observe, that the edge adjacent to a leaf labelled by *a *is optimal in every refinement of *G*, and it has the same type as in *G *(i.e., it is an empty edge). The optimal duplication cost equals 1. The optimal edges with this cost are marked in gene trees *G*_19_, *G*_23 _and *G*_25_. The bottom-right part of this figure depicts the embedding of the optimal rooting of *G*_25 _into the species tree *S *from Figure 3.

**Figure 5:**
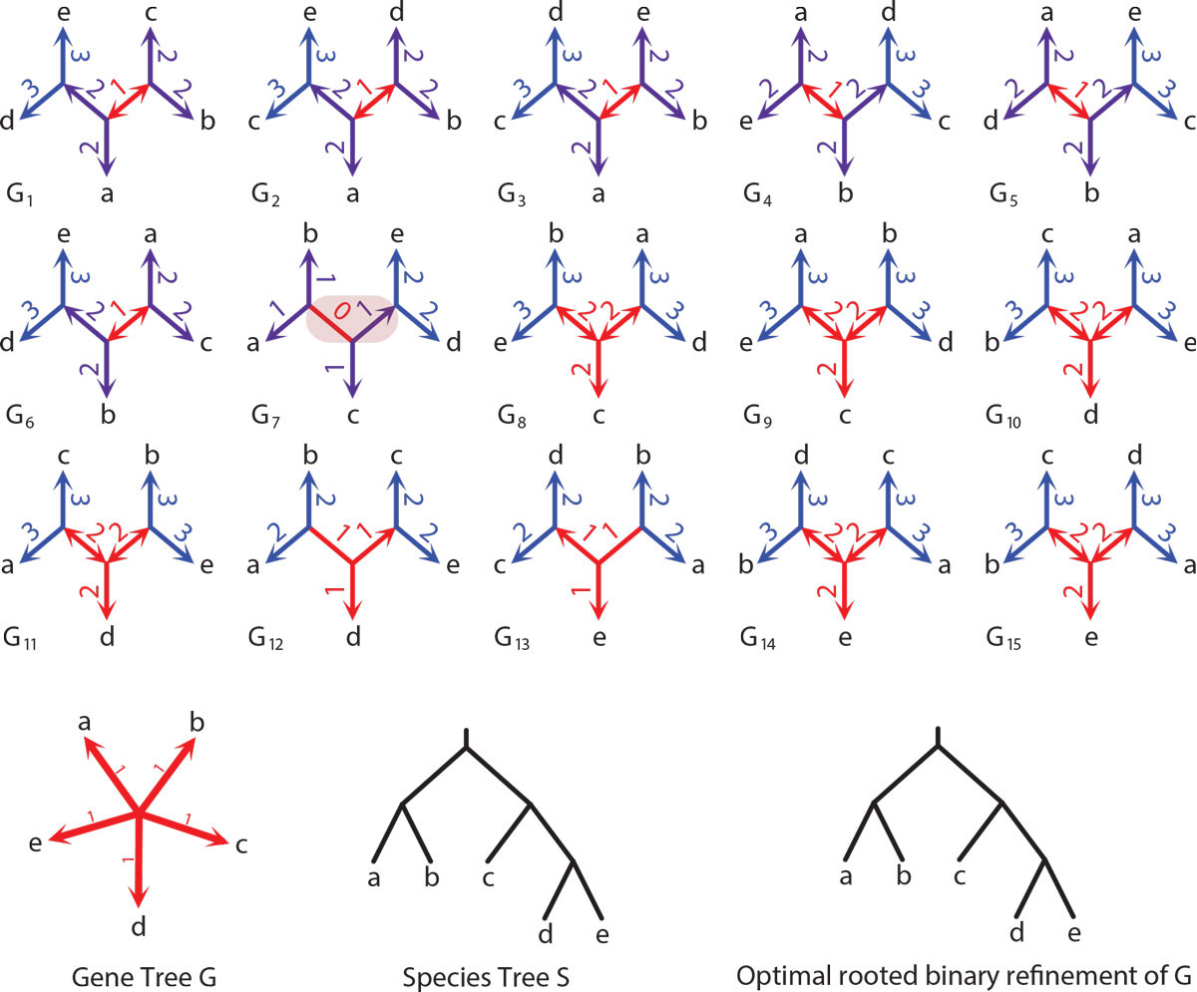
**Star M6 and binary refinements**. The special case of a refinement when the star M6 is present in a gene tree. An optimal edge can be found after rooting at the center node of star M6 and then applying the refinement procedure for rooted gene trees (see Algorithm 1). An optimal edge of every binary refinement of *G *is "surrounded" by the edges related to the star M6 present in *G*. For example, the candidates are two internal edges of *G_i _*for each *i*. The optimal binary refinement of *G *has the gene duplication cost equal to 0 and it is obtained by rooting *G*_7 _at the left internal edge. See also bottom part of this figure. Clearly, it has the same topology as the species tree. Similarly to Figure 3, each edge *e *of the gene tree *G *is decorated with the duplication cost *D*(*G_e_, S*), where *G_e _*is a (not refined) rooting of *G*.

*Proof *The correctness of Algorithm 1 follows from the property that the refinement operation will not change the labels of an existing edge in $\hat{G}$ and properties of stars for binary trees [63]. We analyze the cases from Proposition 1. (i) If *G *has a double edge *e*, then in every (unrooted) binary refinement of *G e *is a double edge. Thus, by Proposition 1 *e *is optimal in every binary refinement of *G*. We conclude that rooting *G *at *e *and removing polytomies from *G_e _*by applying the solution for rooted trees will infer an optimal rooted refinement of *G*. (ii) The same result applies when *G *has an empty edge. (iii) When *G *has only single edges, then the elements of the unique star M6 in *G *are optimal edges in *G*. Similarly, to previous cases these single edges will be present in any (unrooted) binary refinement of *G *(see Figures 3, 4, 5 for example). However, by Proposition 2 and Proposition 1 they are not necessarily optimal in such refinements. To address this problem, observe that any binary unrooted refinement of *G *will have either empty or double edges "surrounded" by the edges previously present in the star of type M6. Thus, we can simply root *G *at the center of the star M6 and then proceed with the refinement procedure for rooted trees. Clearly, the refinement procedure, will infer a rooted gene tree *T *such that its unrooted variant is a binary refinement of *G *with the minimal duplication cost. An example of a gene tree with star M6 with all binary refinements is depicted in Figure 5.

In summary, it is sufficient to identify an optimal edge in *G*, and then proceed accordingly with the refinement procedure. In steps 3-5 the algorithm is evaluating labels of edges from $\hat{G}$. The optimal edge is found in the loop present in steps 6-7. Finally, the refinement procedure is called in steps 9-10 depending on the type of the star.    □

**Theorem 3 ***Algorithm 1 requires O*(*|G||S|*) *time, while the reduction (steps 1-7) can be completed in O*(*|G| *+ *|S|*) *time and space*.

*Proof *As desired, the result follows from [44] and [37].

**Algorithm 1 **Resolving polytomies in unrooted gene trees

1: **Input **A binary species tree *S*, an unrooted gene tree *G *with at least three leaves *L*(*G*) *⊆ L*(*S*).

2: **Output **The rooted binary refinement of *G *with the minimal duplication cost.

3: **Let ***m_x_,y *be the label (a node from *S*) of 〈*x, y*〉 in $\hat{G}$. *// can be computed in O*(*|G|*) *steps *[44].

4: **Let ***v *be a node from *VG*.

5: **Let ⊤ **:= *m_v_,_w _*⊕ *m_w,v _*for some edge 〈*v, w*〉 in *G*.

6: **While **there exists a node *w *adjacent with *v *such that *mw,v *= ⊤ ≠ *I*= *m_v,w_*

7:    **do: **set *v *:= *w *(star M1).

8: **f ***v *is incident with a empty/double edge 〈*v, w*〉, that is, *m_v,w _*= ⊤ = *m_w,v _*or *m_v,w _≠ *⊤≠ *m_w,v_*

9:    **then return ***Bin*(*G*_〈*v,w*〉_*, S*) (optimal edge found in star M2-M5)

10:   **else return ***Bin*(*G_v_, S*) (*v *is the center of star M6).

Examples of (unrooted) binary refinements with costs of all rootings of an unrooted gene tree with multifurcations are depicted in Figures 3, 4 and 5.

### Polytomies and other cost functions

Similarly to the gene duplication cost we show results for other cost functions that are related to the duplication cost [63]. Here, we introduce for the first time a general approach, similar to [32,44], for the case where both trees, i.e., a gene tree and a species tree can be non-binary.

Costs can be defined for rooted trees as follows:

$$\rho_{K}\left(T,S\right)=\underset{g\in I\left(T\right)}{\sum }\xi_{K}\left(g\right),$$

where *T *is a rooted gene tree and *S *is a species tree such that *L*(*T *) ⊆ *L*(*S*), *I*(*T *) is the set of all internal nodes of *T , K *is a cost name and *ξ_K _*: *I*(*T *) *→ R *is a *contribution *function that for an internal node *v *of *T *defines a contribution of *v *to the cost *K *when comparing *T *and *S*. For a node *v *in a rooted tree, by *c*(*v*) we denote the cluster of *v *defined as the set of all leaf labels visible from *v*. The contribution functions for standard costs are defined as follows. Let *g *be an internal node of *T *and *M *be the lca-mapping from *T *to *S*.

• Gene duplication (D) cost function: *ξ_D _*(*g*) = 1 if *g *has a child *c *such that *M *(*g*) =*M *(*c*), and *ξ_D _*(*g*) = 0 otherwise.

• Deep coalescence (DC): $\xi_{D}\left(g\right)=\underset{}{\sum }g\prime$, is a child of *_g _||lM *(*g*)*, M *(*g!*)||, where *||x, y|| *is the number of edges on the shortest path connecting nodes *x *and *y *in *S*.

• Robinson-Foulds cost (RF): *ξ_RF _*(*g*) = 1 if *c*(*g*) ≠ *c*(*M *(*g*)) and *ξ_RF _*(*g*) = 0 otherwise.

Note that the classical Robinson-Foulds distance can be obtained by *RF *(*T, S*) = *|I*(*S*)*| *+ 2 ∗ *ρRF *(*T, S*) *− |I*(*T *)|. Additionally, we have to assume that for the RF distance *T *is bijectively labelled by the labels present in *L*(*S*). For more details and discussion please refer to [44,63].

For an unrooted gene tree *G*, a species tree *S*, the unrooted cost is defined by:

$$\text{Δ}\left(G,S,f\right)=\underset{e\in E_{G}}{\text{min}}f\left(e\right),$$

where *f *: *E_G _→ R *is a cost function usually defined for a cost *K *by *f*(*e*) = *ρ_K _*(*G_e_, S*). Assume that *f_S_*(*e*) = *D*(*G_e_, S*), then it can be proved that *ur D*(*G, S*) = Δ(*G, S, f_S_*).

In the previous section we described the solution to Problem 1 defined for the duplication cost by reducing the unrooted problem to a rooted one in linear time and space. Here, we show that the same kind reduction can by applied for the DC and RF cost functions.

**Problem 2 **(Unrooted refinement under DC cost) *For a given unrooted gene tree G and a binary species tree S, find a binary refinement under all rootings of G that minimizes the DC cost*.

**Problem 3 **(Unrooted refinement under RF cost) *For a given unrooted gene tree G and a binary species tree S, find a binary refinement under all rootings of G that minimizes the RF cost*.

The result for the DC and the RF cost follows from [32] (Proposition 1 and Proposition 2) and [44] (Proposition 1 and Proposition 2), respectively. We conclude, that the statement from Theorem 1 also holds for the DC and RF cost functions. Therefore, Algorithm 1 can be used for locating an optimal edge or star M6 in an unrooted gene tree with multifurcations. Then after such a rooting is identified, one can apply the solution that removes polytomies from rooted gene trees. Clearly this reduction can be performed in linear time and space for both cost functions.

**Problem 4 **(Rooted refinement under DC cost) *For a given rooted gene tree G and a binary species tree S, find a binary refinement under all rootings of G that minimizes the DC cost*.

**Problem 5 **(Rooted refinement under RF cost) *For a given rooted gene tree G and a binary species tree S, find a binary refinement under all rootings of G that minimizes the RF cost*.

According to our knowledge Problem 4 and Problem 5 are open, with the exception that Problem 4 can be solved in quadratic time for the case when the gene tree has a bijective leaf labelling [36]. We conjecture that these two problems can be solved in polynomial time similarly to the problem under the duplication cost [35] (see *Bin*(*Ge, S*) in Algorithm 1). Our reduction shows that Problem 2 and Problem 3 have the same time complexity as the rooted ones.

## Conclusion

To deal with discordance in practice we introduced a binary refinement model for the well-studied Robinson-Foulds, duplication, and deep coalescence costs. To compute these costs we described novel linear time reductions, from which quadratic time algorithms follow for the duplication cost and for the deep coalescence cost when constrained to bijective labelings. Our binary refinement model together with the efficient algorithms allows the exploitation of the full range of available gene trees. Finally, our algorithms not only compute optimal binary refinement costs efficiently, but also simultaneously root and refine gene trees optimally. However, the time complexity of the Robinson-Foulds cost for unrooted and non-binary gene trees will depend on the time complexity of computing this cost for rooted non-binary gene trees, which is unknown to the best knowledge of the authors.

## Competing interests

The authors declare that they have no competing interests.

## Authors' contributions

PG and OE contributed equally to the writing of the paper. Both authors read and approved the final manuscript.

## Declarations

This work was conducted as a part of the Gene Tree Reconciliation Working Group at the National Institute for Mathematical and Biological Synthesis, sponsored by the U.S. National Science Foundation, the U.S. Department of Homeland Security, and the U.S. Department of Agriculture through NSF Award #EF-0832858, with additional support from The University of Tennessee, Knoxville. Partial support was provided to OE by the NSF (#0830012 and #106029), and to PG and OE by NCN #2011/01/B/ST6/02777.

This article has been published as part of *BMC Bioinformatics *Volume 15 Supplement 13, 2014: Selected articles from the 9th International Symposium on Bioinformatics Research and Applications (ISBRA'13): Bioinformatics. The full contents of the supplement are available online at http://www.biomedcentral.com/bmcbioinformatics/supplements/15/S13.

## References

[B1] Avise JC Molecular Markers, Natural History, and Evolution 2004 2 Sinauer Associates, Sunderland, MA

[B2] Felsenstein J Inferring Phylogenies 2004 Sinauer Associates, Sunderland, MA

[B3] Arnason U Adegoke JA Bodin K Born EW Esa YB Gullberg A Nilsson M Short RV Xu X Janke A Mammalian mitogenomic relationships and the root of the eutherian tree Proc Natl Acad Sci USA 2002 99 12 8151 6 10.1073/pnas.102164299 12034869 PMC123036

[B4] Ishiguro NB Miya M Nishida M Basal euteleostean relationships: a mitogenomic perspective on the phylogenetic reality of the "protacanthopterygii" Mol Phylogenet Evol 2003 27 3 476 88 10.1016/S1055-7903(02)00418-9 12742752

[B5] Phillips MJ Penny D The root of the mammalian tree inferred from whole mitochondrial genomes Mol Phylogenet Evol 2003 28 2 171 85 10.1016/S1055-7903(03)00057-5 12878457

[B6] Douglas DA Gower DJ Snake mitochondrial genomes: phylogenetic relationships and implications of extended taxon sampling for interpretations of mitogenomic evolution BMC Genomics 2010 11 14 20055998 10.1186/1471-2164-11-14 PMC2820454

[B7] Floudas D Binder M Riley R Barry K Blanchette RA Henrissat B Martínez AT Otillar R Spatafora JW Yadav JS Aerts A Benoit I Boyd A Carlson A Copeland A Coutinho PM de Vries RP Ferreira P Findley K Foster B Gaskell J Glotzer D Górecki P Heitman J Hesse C Hori C Igarashi K Jurgens JA Kallen N Kersten P Kohler A Ku¨es U Kumar TKA Kuo A LaButti K Larrondo LF Lindquist E Ling A Lombard V Lucas S Lundell T Martin R McLaughlin DJ Morgenstern I Morin E Murat C Nagy LG Nolan M Ohm RA Patyshakuliyeva A Rokas A Ruiz-Duen˜as FJ Sabat G Salamov A Samejima M Schmutz J Slot JC St John F Stenlid J Sun H Sun S Syed K Tsang A Wiebenga A Young D Pisabarro A Eastwood DC Martin F Cullen D Grigoriev IV Hibbett DS The paleozoic origin of enzymatic lignin decomposition reconstructed from 31 fungal genomes Science 2012 336 6089 1715 9 10.1126/science.1221748 22745431

[B8] Sjölander K Phylogenomic inference of protein molecular function: advances and challenges Bioinformatics 2004 20 2 170 9 10.1093/bioinformatics/bth021 14734307

[B9] McCormack JE Hird SM Zellmer AJ Carstens BC Brumfield RT Applications of next-generation sequencing to phylogeography and phylogenetics Mol Phylogenet Evol 2013 66 2 526 38 10.1016/j.ympev.2011.12.007 22197804

[B10] Pamilo P Nei M Relationships between gene trees and species trees Molecular biology and evolution 1988 5 5 568 583 3193878 10.1093/oxfordjournals.molbev.a040517

[B11] Doyle JJ Gene trees and species trees: molecular systematics as one-character taxonomy Systematic Botany 1992 144 163

[B12] Maddison WP Gene trees in species trees Systematic biology 1997 46 3 523 536 10.1093/sysbio/46.3.523

[B13] Ballard JWO Rand DM The population biology of mitochondrial dna and its phylogenetic implications Annual Review of Ecology, Evolution, and Systematics 2005 621 642

[B14] Philippe H Brinkmann H Lavrov DV Littlewood DTJ Manuel M Wörheide G Baurain D Resolving difficult phylogenetic questions: why more sequences are not enough PLoS Biol 2011 9 3 1000602 10.1371/journal.pbio.1000602 PMC3057953 21423652

[B15] Ohno S Evolution by Gene Duplication 1970 Springer, Berlin

[B16] Lynch M Conery JS The evolutionary fate and consequences of duplicate genes Science 2000 290 5494 1151 5 10.1126/science.290.5494.1151 11073452

[B17] Chojnowski JL Kimball RT Braun EL Introns outperform exons in analyses of basal avian phylogeny using clathrin heavy chain genes Gene 2008 410 1 89 96 10.1016/j.gene.2007.11.016 18191344

[B18] Hackett SJ Kimball RT Reddy S Bowie RCK Braun EL Braun MJ Chojnowski JL Cox WA Han KL Harshman J Huddleston CJ Marks BD Miglia KJ Moore WS Sheldon FH Steadman DW Witt CC Yuri T A phylogenomic study of birds reveals their evolutionary history Science 2008 320 5884 1763 8 10.1126/science.1157704 18583609

[B19] Page RDM Charleston MA Reconciled trees and incongruent gene and species trees DIMACS Series in Discrete Mathematics and Theoretical Computer Sciences 1997 37

[B20] Maddison WP Reconstructing character evolution on polytomous cladograms Cladistics - The International Journal of the Willi Hennig Society 1989 5 4 365 377 10.1111/j.1096-0031.1989.tb00569.x 34933477

[B21] Górecki P Burleigh JG Eulenstein O Maximum likelihood models and algorithms for gene tree evolution with duplications and losses BMC Bioinformatics 2011 12 Suppl 1 15 10.1186/1471-2105-12-S1-S15 21342544 PMC3044269

[B22] Bininda-Emonds ORP Phylogenetic Supertrees 2004 Springer, Berlin

[B23] Bryant D Tsang J Kearney PE Li M Computing the quartet distance between evolutionary trees Symposium on Discrete Algorithms 2000 285 286

[B24] Strimmer K von Haeseler A Quartet puzzling: A quartet maximum likelihood method for reconstructing tree topologies Molecular Biology and Evolution 1996 13 964 969 10.1093/oxfordjournals.molbev.a025664

[B25] DasGupta B He X Jiang T Li M Tromp J Zhang L On distances between phylogenetic trees SODA 1997 427 436

[B26] Bordewich M Semple C On the computational complexity of the rooted subtree prune and regraft distance Annals of Combinatorics 2004 8 409 423

[B27] Zheng Y Zhang L Are the duplication cost and the robinson-foulds distance equivalent? J Comput Biol(accepted) 24988427 10.1089/cmb.2014.0021 PMC4116105

[B28] Wu YC Rasmussen MD Bansal MS Kellis M Treefix: statistically informed gene tree error correction using species trees Syst Biol 2013 62 1 110 20 10.1093/sysbio/sys076 22949484 PMC3526801

[B29] Gordon JB Bansal MS Eulenstein O Vision TJ Zhang, A., Borodovsky, M., Özsoyoglu, G., Mikler, A.R. Inferring species trees from gene duplication episodes BCB 2010 ACM, New York, NY, USA 198 203

[B30] Sanderson MJ McMahon MM Inferring angiosperm phylogeny from EST data with widespread gene duplication BMC Evolutionary Biology 2007 7 Suppl 1 S3 10.1186/1471-2148-7-S1-S3 17288576 PMC1796612

[B31] Eulenstein O Predictions of gene-duplications and their phylogenetic development 1998 PhD thesis, University of Bonn, Germany GMD Research Series No. 20 / 1998, ISSN: 1435-2699

[B32] Górecki P Eulenstein O Deep coalescence reconciliation with unrooted gene trees: Linear time algorithms LNCS 2012 7434 531 542

[B33] Bansal AK Meyer TE Evolutionary analysis by whole-genome comparisons Journal of Bacteriology 2002 184 8 2260 2272 10.1128/JB.184.8.2260-2272.2002 11914358 PMC134949

[B34] Page RDM Holmes EC Molecular Evolution: a Phylogenetic Approach Blackwell Science 1998

[B35] Lafond M Swenson KM El-Mabrouk N An optimal reconciliation algorithm for gene trees with polytomies WABI 2012, LNCS/LNBI 2012 7534 106 122

[B36] Yu Y Warnow T Nakhleh L Algorithms for mdc-based multi-locus phylogeny inference: beyond rooted binary gene trees on single alleles J Comput Biol 2011 18 11 1543 59 10.1089/cmb.2011.0174 22035329 PMC3216099

[B37] Zheng Y Wu T Louxin Z Reconciliation of gene and species trees with polytomies 2012 eprint arXiv:1201.3995v2 [q-bio.PE]

[B38] Robinson DF Foulds LR Comparison of phylogenetic trees Mathematical Biosciences 1981 53 131 147 10.1016/0025-5564(81)90043-2

[B39] Semple C Steel MA Phylogenetics. Oxford Lecture Series in Mathematics and Its Applications 2003 Oxford University Press, USA (Book 24)

[B40] Felsenstein J Inferring Phylogenies 2004 Sinauer Associates, Sunderland, Mass

[B41] Mecham CA Felsenstein, J. Theoretical and computational considerations of the compatibility of qualitative taxonomic characters 1983 1 Springer, Berlin 304 314 NATO ASI Series

[B42] Day WHE Optimal algorithms for comparing trees with labeled leaves Journal of Classification 1985 2 1 7 28 10.1007/BF01908061

[B43] Pattengale ND Gottlieb EJ Moret BME Efficiently computing the robinson-foulds metric J Comput Biol 2007 14 6 724 35 10.1089/cmb.2007.R012 17691890

[B44] Górecki P Eulenstein O A Robinson-Foulds measure to compare unrooted trees with rooted trees LNCS 2012 7292 102 114

[B45] Bryant D Steel M Computing the distribution of a tree metric IEEE/ACM Trans Comput Biol Bioinform 2009 6 3 420 6 19644170 10.1109/TCBB.2009.32

[B46] Steel MA Penny D Distributions of tree comparison metrics - some new results Systemtic Biology 1993 42 2 126 141

[B47] Dryer MS Haspelmath M The World Atlas of Language Structures Online 2011 Max Planck Digital Library, Munich

[B48] Alcock J Animal Behavior: An Evolutionary Approach 2005 Sinauer Associates, Sunderland, MA

[B49] Goodman M Czelusniak J Moore GW Romero-Herrera AE Matsuda G Fitting the gene lineage into its species lineage. a parsimony strategy illustrated by cladograms constructed from globin sequences Systematic Zoology 1979 28 132 163 10.2307/2412519

[B50] Maddison WP Gene trees in species trees Syst Biol 1997 46 523 536 10.1093/sysbio/46.3.523

[B51] Zhang L On a Mirkin-Muchnik-Smith conjecture for comparing molecular phylogenies Journal of Computational Biology 1997 4 2 177 187 10.1089/cmb.1997.4.177 9228616

[B52] Ma B Li M Zhang L On reconstructing species trees from gene trees in term of duplications and losses RECOMB 1998 182 191

[B53] Page RDM Extracting species trees from complex gene trees: reconciled trees and vertebrate phylogeny Molecular Phylogenetics and Evolution 2000 14 89 106 10.1006/mpev.1999.0676 10631044

[B54] Cotton JA Page RDM Going nuclear: gene family evolution and vertebrate phylogeny reconciled P Roy Soc Lond B Biol 2002 269 1555 1561 10.1098/rspb.2002.2074 PMC1691073 12184825

[B55] Martin AP Burg TM Perils of paralogy: using hsp70 genes for inferring organismal phylogenies Syst Biol 2002 51 4 570 87 10.1080/10635150290069995 12228000

[B56] McGowen MR Clark C Gatesy J The vestigial olfactory receptor subgenome of odontocete whales: phylogenetic congruence between gene-tree reconciliation and supermatrix methods Syst Biol 2008 57 4 574 90 10.1080/10635150802304787 18686195

[B57] Katz LA Grant JR Parfrey LW Burleigh JG Turning the crown upside down: gene tree parsimony roots the eukaryotic tree of life Syst Biol 2012 61 4 653 60 10.1093/sysbio/sys026 22334342 PMC3376375

[B58] Plachetzki DC Degnan BM Oakley TH The origins of novel protein interactions during animal opsin evolution PLoS One 2007 2 10 1054 10.1371/journal.pone.0001054 PMC2013938 17940617

[B59] Górecki P Tiuryn J Inferring phylogeny from whole genomes Bioinformatics 2007 23 2 116 122 10.1093/bioinformatics/btl296 17237078

[B60] Chen K Durand D Farach-Colton M NOTUNG: a program for dating gene duplications and optimizing gene family trees J Comput Biol 2000 7 3-4 429 447 10.1089/106652700750050871 11108472

[B61] Chang WC Phylogenetic reconciliation under gene tree parsimony PhD thesis, Iowa State University 2012

[B62] Eulenstein O Huzurbazar S Liberles DA Reconciling Phylogenetic Trees Evolution after Gene Duplication 2010 John Wiley & Sons, Inc., Hoboken, NJ, USA

[B63] Górecki P Eulenstein O Tiuryn J Unrooted Tree Reconciliation: A Unified Approach IEEE/ACM Transactions on Computational Biology and Bioinformatics 2013 10 2 522 536 23929875 10.1109/TCBB.2013.22

[B64] Górecki P Tiuryn J DLS-trees: a model of evolutionary scenarios Theoretical Computer Science 2006 359 1-3 378 399 10.1016/j.tcs.2006.05.019

[B65] Zheng Y Wu T Zhang L A linear-time algorithm for reconciliation of non-binary gene tree and binary species tree Lecture Notes in Computer Science 2013 8287 190 201 10.1007/978-3-319-03780-6_17

